# Co-Immunization Efficacy of Recombinant Antigens against *Rhipicephalus microplus* and *Hyalomma anatolicum*Tick Infestations

**DOI:** 10.3390/pathogens12030433

**Published:** 2023-03-09

**Authors:** Balasamudram Chandrasekhar Parthasarathi, Binod Kumar, S. K. Bhure, Anil Kumar Sharma, Manisha, Gaurav Nagar, Sachin Kumar, Abhijit Nandi, Haranahally Vasanthachar Manjunathachar, Gajanan M. Chigure, Mukesh Shakya, Muthu Sankar, José de la Fuente, Srikant Ghosh

**Affiliations:** 1 Entomology Laboratory, Division of Parasitology, ICAR-Indian Veterinary Research Institute, Izatnagar, Bareilly 243122, India; 2 Department of Veterinary Parasitology, College of Veterinary Science & Animal Husbandry, Kamdhenu University, Junagadh 362001, India; 3 Division of Biochemistry, ICAR-Indian Veterinary Research Institute, Izatnagar, Bareilly 243122, India; 4 Department of Veterinary Parasitology, Faculty of Veterinary & Animal Sciences, West Bengal University of Animal & Fishery Sciences, Kolkata 700037, India; 5 ICMR-National Animal Resource Facility for Biomedical Research (NARFBR), Hyderabad 500078, India; 6 Department of Parasitology, College of Veterinary and Animal Sciences, Maharashtra Animal and Fishery Sciences University, Parbhani 431402, India; 7 Department of Veterinary Parasitology, College of Veterinary Science & Animal Husbandry, Nanaji Deshmukh Veterinary Science University, Mhow 453446, India; 8 SaBio, Instituto de Investigación en Recursos Cinegéticos IREC (CSIC-UCLMJCCM), 13005 Ciudad Real, Spain; 9 Department of Veterinary Pathobiology, Center for Veterinary Health Sciences, Oklahoma State University, Stillwater, OK 74078, USA

**Keywords:** *Rhipicephalus microplus*, *Hyalomma anatolicum*, Bm86, tropomyosin, subolesin, co-vaccination

## Abstract

The immunoprophylactic management of ticks is the most effective option to control tick infestations and counter spread the acaricide resistance problem worldwide. Several researchers reported an inconsistent efficacy of the single antigen-based immunization of hosts against different tick species. In the present study, to develop a multi-target immunization protocol, proteins from *Rhipicephalus microplus* BM86 and *Hyalomma anatolicum* subolesin (SUB) and tropomyosin (TPM) were targeted to evaluate the cross-protective potential. The sequence identities of the *BM86*, *SUB*, and *TPM* coding genes amongst Indian tick isolates of targeted species were 95.6–99.8%, 98.7–99.6%, and 98.9–99.9%, respectively, while at the predicted amino acid level, the identities were 93.2 to 99.5, 97.6 to 99.4, and 98.2 to 99.3%. The targeted genes were expressed in the eukaryotic expression system, pKLAC2-*Kluyveromyces lactis*, and 100 µg each of purified recombinant protein (Bm86-89 kDa, SUB-21 kDa, and TPM-36 kDa) mixed with adjuvant was injected individually through the intramuscular route at different sites of the body on days 0, 30, and 60 to immunize cross-bred cattle. Post-immunization, a statistically significant (*p* < 0.001) antibody response (IgG, IgG1, and IgG2) in comparison to the control, starting from 15 to 140 days, against each antigen was recorded. Following multi-antigen immunization, the animals were challenged twice with the larvae of *R. microplus* and *H. anatolicum* and theadults of *H. anatolicum*, and a significant vaccine efficacy of 87.2% and 86.2% against *H. anatolicum* larvae and adults, respectively, and 86.7% against *R. microplus* was obtained. The current study provides significant support to develop a multi-antigen vaccine against cattle tick species.

## 1. Introduction

Livestock rearing is an integral component of the Indian agriculture system and plays a significant role in the sustainable maintenance of the economy and for nutritional security. Of the total production in the agriculture sector of India, livestock alone contributes 5.1% of GDP [1]. Ticks are considered to be second worldwide to mosquitoes as vectors of human diseases [2] and are regarded as the most important arthropod-borne disease of livestock, humans, and companion animals [2,3]. Tick as an obligate blood feeder of vertebrate hosts, act as vectors of different pathogens such as protozoa, virus, bacteria/rickettsia, fungi, and filaroids [4,5,6]. They can even cause allergies, irritation, paralysis, and toxicosis in animals [7,8]. Amongst the 106 tick species infesting Indian animals, *Rhipicephalus microplus* and *Hyalomma anatolicum* are the most widely distributed and economically important species, transmitting *Babesia bigemina*, *Anaplasma marginale*, *Theileria annulata*, *Theileria buffeli*, and *Theileria lestoquardi* [9]. A number of tick-borne zoonotic diseases, viz., Indian tick typhus (ITT), Kyasanur Forest Disease (KFD), and Crimean Congo Hemorrhagic fever (CCHF), of humans are frequently reported from India [9,10,11] and other parts of the world [12,13] and are spreading [14,15].

Tick management in most countries heavily relies on acaricide treatment with partial success [16,17]. Continuous use of acaricides has resulted in the selection of resistant populations of ticks and has been reported from different parts of the world [18,19,20]. There is also evidence that presents that strategies based on the use of acaricides for tick management are not cost effective [21,22,23]. In India and other parts of the world, reports of acaricide resistance have increased significantly during the last ten years, and tick resistance to synthetic pyrethroids (deltamethrin, cypermethrin, and fenvalerate), organophosphates (diazinon and malathion), amidines (amitraz), and macrocyclic lactones (ivermectin) has been reported [24,25]. In addition to resistance, the continuous and indiscriminate use of chemical acaricides has led to increases in the level of environmental pollutants and the contamination of milk and meat products with drug residues [26].

Alternatively, the immunological control of ticks is considered promising and sustainable [27]. The development of vaccines using multiple antigens that could target a broad range of tick species may also prevent or reduce the transmission of pathogens [28,29,30,31]. Generally, the ‘concealed antigen’ is targeted for the development of anti-tick vaccines because ticks may not be in a position to evolve counter effects to host immune systems as in the case of ‘exposed antigens’ [28]. However, the vaccine commercialized using concealed antigens provided variable efficacy against homologous and heterologous tick species [29]. The variable efficacy of the commercial vaccine against different strains of *R. microplus* warrants the identification of novel molecules, and accordingly, few potential molecules were identified [30,31,32]. It was assumed that antibodies would enter into the body of ticks through blood meal, bind to the targeted organs, and disrupt vital functions leading to the death of the ticks. Some studies also showed that the host immunoglobulins can cross the gut of ticks to the hemolymph to cells [33,34]. These findings have increased the possibility of targeting both intracellular and secretary molecules in vaccine development. Bm86, tropomyosin (TPM), and subolesin (SUB) are proteins located in gut cells (digest cells), muscles fibres, and in other organs and are involved in physiological and cellular functions [35,36,37]. These proteins are conserved among different tick species and are used in different immunization formats, and a differential response to challenge infestations was reported [35,36,37,38,39,40], for example, 45% and 25% protection against homologous and heterologous challenges using the Bm86 vaccine [35], 41–65% against homologous and 54% against heterologous challenges using SUB [36,41], and 66% protection against homologous challenges using the TPM [37] of *R. microplus* and/or *H. anatolicum*. To increase vaccine efficacy, cocktails of antigens comprising two to three antigens were attempted, and the synergistic and antagonistic effects of antigens in vaccines were also reported [40]. In contrast to cocktails of antigens, multiple antigens injected separately may elicit a better response as the chance of interference of one antigen with other can be avoided [41,42,43,44]. The selection of the proteins was based on the previous results using these antigens. The immunization potential of SUB and TPM antigens was thoroughly evaluated previously in our laboratory, and a significant efficacy against challenge infestations using single antigens was noted [36,37,41]. The selection of Bm86 of *R. microplus* was based on the efficacy reported by different researchers [45,46,47,48]. Unlike other countries, Indian cattle are infested predominantly with *R. microplus* and *H. anatolicum*, transmitting fatal pathogens. To have significant protection against both species of ticks, the present study was targeted using multiple antigens from both species. Accordingly, in the present study, three functionally active proteins, Bm86 of the Indian strain of *R. microplus* and SUB and TPM of *H. anatolicum*, were targeted for the development of an immunization protocol against the two most economically important tick species.

## 2. Materials and Methods

### 2.1. Ticks and Experimental Animals 

*R. microplus* (IVRI-I) and *H. anatolicum* (IVRI-II) maintained in the Entomology laboratory, Division of Parasitology, were used as reference materials. Cross-bred bovine calves (*Bos indicus* × *B. taurus*) above four months of age were procured from the section of livestock production management, IVRI, maintained in the tick-proof shed of the Division of Parasitology as per the standard guidelines of the Committee for the Purpose of Control and Supervision on Experiments on Animals (CPCSEA) [No.F.26-1/ 2018-19/J.D(R) dated 7 October 2019]. Similarly, healthy 1 to 1.5 kg New Zealand white rabbits were procured from the laboratory animal resource section of the institute and maintained in the small animal house of the division. The rabbits were used for rearing *H. anatolicum* and for raising hyper-immune sera. The rabbits were maintained and handled as per the approved guidelines laid down by the Institute Animal Ethics Committee [No.F.26-1/2015-16/ J.D(R) dated 20 February 2019]. Field isolates of *R. microplus* and *H. anatolicum* were collected from different agroclimatic zones of India following the two-step stratified sampling procedure. The collected ticks were washed thoroughly, classified, weighed, labeled, and stored at −80 °C. These ticks were processed for molecular studies.

The challenge study was conducted as per the method of Ghosh and Azhahianambi [49]. In this method, the laboratory reared *R. microplus* and *H. anatolicum* larvae which were released on left and right ear pinna using the ear bag method. The larvae of *H. anatolicum* and the adults of *R. microplus* were collected from the ear bags daily, and entomological data were generated, keeping the dropped ticks at 28 °C and 85% RH in a BOD incubator.

### 2.2. In Vitro Amplification, Cloning, and Sequencing of the Genes

Laboratory-reared reference tick strains, *R. microplus* (IVRI-I) and *H. anatolicum* (IVRI-II), were used for isolation of RNA and the subsequent amplification of the targeted genes. For each gene, three engorged female ticks were randomly picked from the reference tick strains, (IVRI-I) and (IVRI-II), and were weighed and stored at −80 °C. The ticks were triturated in 2 mL of Trizol^®^ (Thermo Fisher Scientific, Waltham, MA, USA) reagent (1 mL/100 mg tissue). The tick lysate was centrifuged at 10,000 rpm for 5 min; the supernatant was mixed with chloroform (0.2 mL/1 mL of Trizol) and incubated at room temperature after vigorous shaking. The aqueous phase was mixed with absolute isopropyl alcohol and centrifuged at 13,000 rpm for 15 min at 4 °C. The supernatant was discarded, and the pellet was washed with 70% ethanol. The RNA pellet was air dried and allowed to dissolve in DEPC-treated water after heating the tube at 55 °C for 15 min. The RNA was stored at −80 °C. The cDNA was synthesized from the extracted RNA (500 ng^−1^μg) using a first-strand cDNA synthesis kit (Thermo Fisher Scientific, Waltham, MA, USA) following the manufacturer’s instructions. The PCR primers were designed for the amplification of the targeted genes (Table S1). The PCR mixture of 25 µL was set up comprising 2.5 µL of 10 × Dream Taq Green Buffer, 0.5 of µL dNTPs (10 mM), 1 µL of each of the forward and reverse primers (10 µM), 0.3 µL of DreamTaq DNA polymerase (5 U/µL), 2 µL of magnesium chloride (25 mM MgCl_2_), 2 µL of cDNA (50 ng), and nuclease-free water (NFW). The reaction conditions were an initial denaturation at 94 °C for 5 min followed by 32 cycles of denaturation at 94 °C for 30 s, annealing at 52 °C for 30 s for *BM86* and *SUB* and 58 °C for 40 s for *TPM*, and extension at 72 °C for 1 min. The final extension was kept at 72 °C for 10 min. The amplicon was purified, cloned in T/A cloning vector (pTZ57R/T) using an InsTAclone PCR cloning kit (Thermo Scientific, Waltham, MA, USA), and sequenced.

### 2.3. Sequence Homology of Targeted Genes of R. microplus and H. anatolicum

Field isolates of *R. microplus* (69) and *H. anatolicum* (15) were collected from nine states of India (Table S2). A pool of 100–150 ticks/district were collected, identified, and cleaned thoroughly. cDNA was prepared from the total RNA, and the genes were amplified at optimized PCR conditions, cloned, and sequenced. A minimum of three clones of each strain were sequenced. Nucleotides and their deduced amino acid sequences from different Indian field isolates of *R. microplus*, *H. anatolicum*, other tick species, arthropods, and mammals were aligned and analysed in silico using BioEdit software (Version 7.0.5.3), Clustal*W*, and Mega version 10. The coding sequences (CDS) of the selected proteins (Bm86, SUB, and TPM) of different Indian field isolates were downloaded. The amino acid sequences of each protein were aligned using the Clustal*W* tool. The aligned sequences were processed through BioEdit software (Version 7.0.5.3), and entropy plots were calculated for each protein. 

### 2.4. Relative Gene Expression Profile

The expression profiling of the *SUB* and *TPM* genes in different stages of *H. anatolicum* was previously standardized in the laboratory [36,37]. To study the expression profile of the *Bm86* gene in different stages, total RNA was isolated from three biological replicates of eggs, larvae, engorged nymphs, partially fed females, partially fed males, fed males, and engorged females of *R. microplus (*IVRI-I), and cDNA was prepared. The housekeeping genes, *EF 1-α* and *GAPDH*, were used as endogenous controls for the relative quantification of gene-specific transcriptome [34]. Using gene-specific primers (Table S1), qPCR was performed as per in-house standardized protocols [34,35]. Briefly, the SYBR green chemistry-based reaction was optimized using the StepOnePlus™ Real-Time PCR System (Applied Biosystem, Waltham, MA, USA) in a 10 μL reaction mixture containing 20 ng of cDNA, 2× Fast SYBR^®^ green master mix, and 1 pg of each of the PCR primers. The reaction conditions were an initial denaturation at 95 °C for 20 s followed by 40 cycles of denaturation at 95 °C for 30 s and annealing/extension at 60 °C for 30 s. Melt curve: 60–95 °C (0.3 °C increment), 15 s per step. For each biological sample, three technical replicates were used along with a no-template control (NTC). The relative quantification of the gene expression was conducted following the described method [50]. For the normalization of the gene expression, the geometric means of the C_T_values of *GAPDH* and *EF 1-alpha* were applied. Considering the 100% (±5%) efficiency of the qPCR primer, the 2^−ΔΔCT^ method was used to calculate the fold change in the gene expression in the test sample compared to the control/reference sample. One-way ANOVA and the Tukey test were used to compare the fold change in the gene expression in comparison to the reference stage (larvae) at a *p* < 0.05 level of significance.

### 2.5. Expression of Proteins in the Eukaryotic Expression System

The full-length IVRI-I *Bm86* (orf) gene was amplified at optimized PCR conditions [38]. The predicted mature protein coding sequences for *Bm86*, *SUB*, and *TPM* were expressed in *K. lactis* GG799 competent cells (New England Biolabs Inc., Ipswich, MA, USA) using the expression vector pKLAC2, which produce recombinant proteins along with HA (Hemagglutinin)-tag for affinity purification. For directional cloning, 5′ ends of both the forward and reverse primers were designed (Table S1) with two restriction enzyme (RE) sites not present in the gene sequence but available at multiple cloning sites (MCS) of vector pKLAC2. A Kex protease processing site was inserted in between the α-MF domain and the N-terminal region of three proteins. The targeted length of the genes was amplified from previously cloned plasmid at optimised PCR conditions and purified. For each gene of interest, 0.5 µg of purified PCR product was subjected to double RE digestion (XhoI and NotI for *BM86* and *SUB*; NcoI and BamHI for *TPM*). Similarly, 1 µg of eukaryotic expression vector pKLAC2 (New England Biolabs Inc., Ipswich, MA, USA) was digested with the same set of RE. Both digested genes and vectors were gel purified, and ligation was performed. The PCR products were cloned into respective restriction sites of the pKLAC2 vector in frame with the α-mating factor (α-MF) secretion leader sequence to give pKLAC2-*Bm86*, pKLAC2-*SUB*, and pKLAC2-*TPM* constructs and then were chemically transformed into competent NEB^®^ 5-alpha F’I^q^ *E. coli* cells. The positive clones were identified, the plasmid was isolated, and sequencing of the insert was conducted to confirm the successful insertion of the *Bm86*, *SUB*, and *TPM* sequences into the pKLAC2 cassette.

For the eukaryotic expression of the targeted proteins, competent *K. lactis* GG799 cells (New England Biolabs Inc., Ipswich, MA, USA) were prepared using 1M sorbitol. For the linear expression cassette, the circular pKLAC2-Bm86, pKLAC2-SUB, and pKLAC2-TPM plasmids were digested with SacIIRE. A total of 1µg of linearized DNA (less than 15 µLvolume) was used to transform chemically *K. lactis* competent cells by heat shock at 37 °C for 1 h in a water bath. The cells were pelleted at 7000 rpm for 2 min, suspended in 1 mL of sterile YPD medium, and centrifuged at 7000 rpm for 2 min. The cell pellet in YPD medium was allowed to grow at 30 °C for three to four hours at 300 rpm. The cell suspensions of 10 µL, 50 µL, and 100 µL were taken into 1.5 mL tubes containing 50 µL of deionized water. The mixture in each tube was spread over a YPD agar plate containing 5 mM acetamide, incubated in the inverted position at 30 °C until colonies appeared (4–5 days). For further growth, 10–20 individual colonies were directly transferred on fresh YPD agar plates containing 5 mM acetamide and incubated at 30 °C for 1–2 days. Yeast cells with correctly integrated expression fragments were verified, the positive clones were scraped from the YPD plate, re-suspended in 10 mLof YPD medium, and grown at 30 °C with shaking (250 rpm) for 7 days. In total, 0.2 mL of overnight culture was transferred to 50 mL of medium and kept in a shaker to obtain OD 600 > 30 units/mL. Each day, 1 mL of supernatant from each 50 mL flask was pipetted out and stored at −20 °C for SDS-PAGE profiling. For each gene of interest, a 50 mL culture was set. The culture supernatant was removed by centrifugation at 15,000× *g* for 30 min, and the supernatant was mixed with protease inhibitor cocktail [one tablet complete (Sigma-Aldrich, St Louis, MO, USA) for 50 mL culture]. An anti-HA affinity column was used to purify the recombinant secreted proteins present in the culture supernatant. The sterility of the purified antigens was assured by filtration through membrane filters two times (0.4 micron). Further, the antigens were mixed with two types of media [fluid thioglycollate medium (FTM) and soybean–casein digest medium (SCDM)] separately and kept for 14 days, and no aerobic or anaerobic microorganism growth was observed.

### 2.6. Raising of Hyper-Immune Sera in Rabbits

Three New Zealand white rabbits, 1–1.5 kg each were procured from the laboratory animal resources (LAR) section, IVRI. Each rabbit was subcutaneously injected with 100 μg of the rBm86, rSUB, and rTPM proteins emulsified with Freund’s complete adjuvant (Sigma-Aldrich, St Louis, MO, USA). Two booster doses were given along with Freund’s incomplete adjuvant (Sigma-Aldrich, St Louis, MO, USA) on the 14th and 28th day after the first immunization. Blood was collected on the 21st and 35th day after the first immunization, and serum was isolated. The serum was stored at −20 °C for future use. The health status of the rabbits was monitored before, during, and after immunization. All rabbits were healthy, and no signs of toxicity were observed during or after immunization.

### 2.7. SDS-PAGE and Western Blot

The purity and molecular weight of recombinant proteins (rBm86, rSUB, and rTPM) were determined on 12% SDS-PAGE [51]. The resolved proteins were subsequently transferred to a nitrocellulose membrane using semi-dry blotting apparatus (Atto, Japan) in 50 mM Tris base, 380 mM glycine, 0.1% SDS, and 20% methanol @ 2 Ma/cm2 for 60–90 min. The unbound surface of the membrane was blocked overnight with 5% skimmed milk in PBS (incubation buffer) at 4 °C. The membrane was washed three times with PBS–Tween (0.05%), and final washing was conducted with PBS (pH 7.4). The membrane was probed with HA-tag polyclonal antibodies (Puregene, Valencia, CA, USA) at 1: 3000 dilutions and was developed with diaminobenzidine (DAB) solution (Amresco, Solon, OH, USA) [52,53]. The recombinant proteins (rBm86, rTPM, and rSUB), 5 µg of each protein, were allowed to run in SDS-PAGE. Then, the proteins were transferred onto a nitrocellulose membrane. Sera collected from the immunized group on days 0, 15, 30, 60, 75, 90, 105, and 120 were diluted (1:16,000 and 1:8000) and were probed with the transferred proteins on the membrane using the Bio-RAD mini-protein^®^ II multi-screen apparatus. The membrane was washed after colour development, and an image was recorded. Two sera samples were used as the control, negative control-I (fetal calf serum), and negative control-II (pooled sera sample of control animals) 

### 2.8. Monitoring of Immune Response

Blood samples were collected from each animal during pre-immunization, immunization, and post-immunization periods at different time intervals, serum was separated, and indirect ELISA was optimized [54]. Briefly, each well was coated with 1 μg/mL of rBm86 and rSUB or 2 μg/mL of rTPM with a sera dilution of 1:16,000 for rBM86 and rSUB and 1:8000 for rTPM, and the secondary antibodies, IgG, IgG1, and IgG2, were diluted to 1:10,000. Peroxidase-mediated colour development was measured at 492 nm in an ELISA reader (Tecan Austria GmbH, Grödig, Austria). The mean optical density was calculated for each time point by grouping the control and immunized animals. Analysis of variance was used for comparing the antibody response among groups of the experimental calves and between different days within the same group of calves. Significance at the 5% level (*p <* 0.05) was used to define differences in different parameters.

### 2.9. Immunization and Challenge Study

Nine cross-bred calves were randomly divided in two groups, immunized (*n* = 6) and control (*n* = 3). Sterilized and frozen protein samples were thawed and emulsified thoroughly with an equal volume of Montanide ISA 50V2 ready-to-use adjuvant (Seppic, France). Three antigens with a dose rate of 100 µg (1–2 mL) each were injected individually through the intramuscular route at different sites of the body on days 0, 30, and 60. The control group of animals was inoculated with 1 mL of adjuvant mixed with 1 mL of PBS. Each calf fromthe immunized and control groups was challenged with the larvae and adults of *R. microplus* and *H. anatolicum* as follows:

1. 10th day of the second booster with ten to twelve-day-old larvae hatched from 50 mg eggs of *H. anatolicum* and *R. microplus;*
2. 20th day of the second booster with larvae hatched from 50 mg eggs of *H. anatolicum;*
3. 30th day of the second booster with larvae hatched from 50 mg eggs of *R. microplus* and unfed adults of *H. anatolicum* (15 female + 30 male on each calf).

The ear bags were regularly checked, and dropped larvae and adults were kept in a BOD incubator maintained at 28 °C and 85% relative humidity. Engorged larvae were maintained until moulting to nymphs and engorgedfemales untilthe end ofegg laying. The immunization efficacy was measured based on the reduction in the number of challenged larvae, moulting and rejection percentage, engorgement weight, and adult fertility rate as the standardized formula [35].

### 2.10. Statistical Analysis 

One-way analysis of variance (ANOVA) with post-hoc Tukey HSD (honestly significant difference) was used for comparing gene transcription levels in different tick stages, the mean variation in theentomological data of ticks fed on the experimental group of animals in comparison to the control group of animals, and for the mean antibody responses of calves in the experimental and control groups at different time points. Significance at a 5% level (*p* ≤ 0.05) was used to define differences in different parameters.

## 3. Results

### 3.1. Genetic Homology of the BM86, SUB, and TPM Coding Genes 

The 578, 492, and 901 bp gene fragments of *Bm86*, *SUB*, and *TPM*, respectively, were sequenced. The GenBank accessions of the submitted sequences were MN088493, MN095773-87, MN115793-98, MN319494-98, MN585688-706, and MT503262-82 for *Bm86*; MN047237-40 and MT642564-74 for *SUB;* and MT642575-89 for the *TPM* gene. The 578 bp nucleotide and its deduced 192 amino acid sequence of the *BM86* gene of 68 field isolates revealed 95.6 to 99.8% and 93.2 to 99.5% [42] identities in the nucleotides and amino acid sequences, respectively. Phylogenetic analysis of the sequences with Indian field isolates showed two distinct clades, E and F. The F clade was formed with conserved *BM86* sequences of strains from Campo Grande (Brazil), Mozambique, Thailand, and China. The Indian field isolates (Dausa) were grouped under clade E. Interestingly, the Gujarat sequences were arranged in a group (red-coloured box) (Supplementary Figure S1). Clade E was further subdivided into subclade E1 (consisting of forty-three sequences) and subclade E2 (formed with twenty-two sequences of Indian field isolates and four Thailand sequences) (Supplementary Figure S1). Subclade E1 has 0 nonsense, 8212 synonymous, and 44 nonsynonymous mutations, whereas the E2 subclade has 0 nonsense, 4170 synonymous, and 54 nonsynonymous mutations. The conserved Bm86 Indian isolates showed a minimum (1) to maximum (11) number of different amino acid substitutions/mutations. Analysis of the state-wise share of the total amino acid substitutions revealed that Rajasthan state contributesa maximum share of 20% of the substitutions/mutations, acting as a geographical hotspot for Bm86 mutations. The state-wise share of amino acid mutations (geographical hot spots) in conserved Bm86 is as follows: Rajasthan (20%) > Maharashtra and Haryana (15%) > Madhya Pradesh and Gujarat (12%) > Uttarakhand and Assam (9%) > Uttar Pradesh and Punjab (4%). The sequence identity analysis showed that the IVRI-I Bm86 protein has 6.76% and 7.22% divergence with the Yeerongpilly (TickGARD^TM^) and Camcord (Cuba) (GAVAC) vaccine strains, respectively. A divergence level of more than 2.8% has been reported as a limiting factor in the variation in efficacy of Bm86-based vaccines [55,56]. The Bm86 protein entropy plot was constructed using Indian field isolates (Figure S2A). The plot shows thirty (30) peaks; these peaks (Supplementary Figure S2A) represent variations across the compared sequences (short peaks represent a low level of variation at a specific amino acid level). The highest variability at R132G and the lowest variability at C29R, G32D, T34K, E38G, S47F, V71I, D143G, V162A, and E168D were recorded.

Similarly, the sequence identities of the *SUB* and *TPM* genes of the IVRI II strain with various field isolates of the same species showed 98.8–99.6% and 98.9–99.5% similarity at the nucleotide level and 97.6–99.4% and 98.2 to 99.3% at the amino acid level, respectively. The predicted amino acid sequence similarity for the same genes in other tick species was in the range of 84.8–99.3% and 87.6–99.6%, respectively. Although the homologues of both the genes are reported in mammals, the percentage identity of deduced amino acid sequence was less than 46% with *B. taurus*/*B. indicus* Akirin/*SUB* and less than 58% with *B. taurusTPM1* or *TPM2* of the IVRI II strain. The phylogenetic tree of the deduced amino acid sequences of *SUB* revealed that the Indian field isolates were arranged in separate clades, but the *SUB* genes of other species diverges from the *SUB* of Indian field isolates (Supplementary Figure S3). The SUB protein entropy plot was constructed using *H. anatolicum* Indian field isolates (Supplementary Figure S2B). The results showed amino acid variations at six locations (six peaks) across the aligned sequences. The number of amino acid variations across the aligned sequences were in following order: D11G/R > W12R > D148V > D13G ≈ C112R ≈ E114Q. The TPM protein entropy plot constructed using *H. anatolicum* Indian field isolates (Supplementary Figure S2C) showed amino acid variations at eight locations (eight peaks) across the aligned sequences, and the amino acid variations were observed in descending order as follows: E145G> R182C > E2D > K112R > R21G > V261A ≈ K268E ≈ Y267C.

### 3.2. Expression Profile of the Bm86 Gene in Different Stages of R. microplus

**Figure 1:**
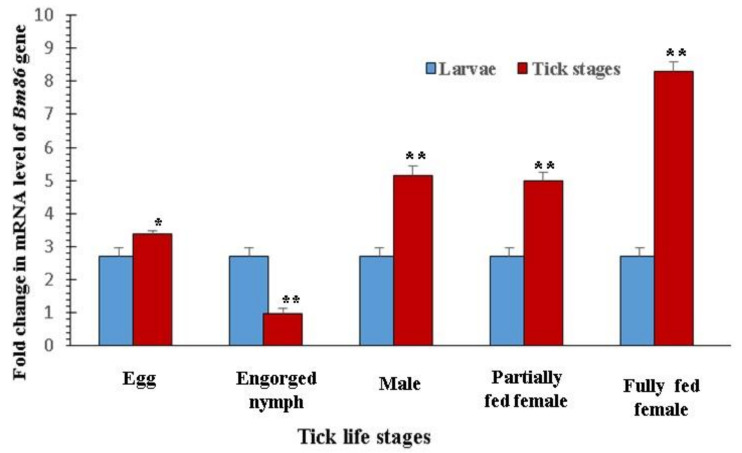
Fold changes in the *Bm86* gene expression at different life stages in comparison to the larvae of *R. microplus* IVRI-I strain [* *p <* 0.05; ** *p <* 0.0001].

The expression profiling of the *SUB* and *TPM* genes of *H. anatolicum* has already been standardized in the laboratory [34,35]. The primer efficiency and R^2^ values of *EF 1-α*, *GAPDH*, and *BM86* were within the normal ranges of 96% to 99.2% and 0.996 to 0.999, respectively. Significantly higher-fold changes in the expression of the *BM86* gene in eggs (*p <* 0.05), males (*p <* 0.0001), partially fed females (*p <* 0.0001), and fully fed females (*p <* 0.0001) were observed in comparison to larvae (Figure 1). 

### 3.3. Expression of Recombinant Proteins

**Figure 2:**
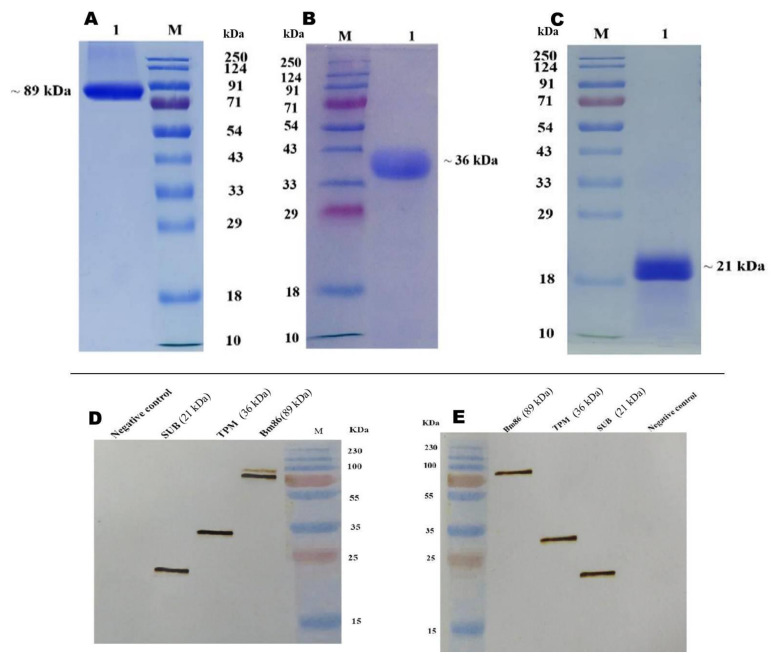
SDS-PAGE profile of the purified rBm86 protein of *R. microplus* IVRI-I (**A**), rTPM (**B**), and rSUB (**C**) protein of *H. anatolicum* IVRI-II; western blot profile of three purified proteins probed with anHA-tag polyclonal antibody (**D**); hyper-immune sera raised in rabbits (**E**); three-colour protein weight marker (Puregene, USA).

The full-length sequence of the *Bm86* gene is 1953 bp. The sequencing of recombinant clones confirms the right-direction cloning of the coding sequence of *Bm86* (1947 bp), *SUB* (492 bp), and *TPM* (855 bp) genes into the pKLAC2 vector. The anticipated linearization of pKLAC2-*Bm86*, pKLAC2-*SUB*, and pKLAC2-*TPM* was observed upon *SacII* RE digestion. The predicted amplification of cloned genes with expression-specific primers was observed in genomic DNA isolated from positively transformed *K. lactis*. Similar levels of the expression of recombinant proteins with a peak on day 4 were observed in all the selected clones. The secreted recombinant proteins, rBm86, rHaTPM, and rHaSUB, were purified from culture media and resolved as 89 kDa, 36 kDa, and 21 kDa, respectively (Figure 2A–C). Upon scaling up production, the batch-wise recovery of rBm86, rHaTPM, and rHaSUB was consistent in the range of 1–2, 2–2.5, and 2.3–3 g/L of culture, respectively. The purity of the purified proteins was more than 90% (Figure 2A–C). rBm86, rHaTPM, and rHaSUB positively reacted with HA-tag polyclonal antibodies and specific hyper-immune sera (Figure 2D). 

### 3.4. Antibody Responses

**Figure 3:**
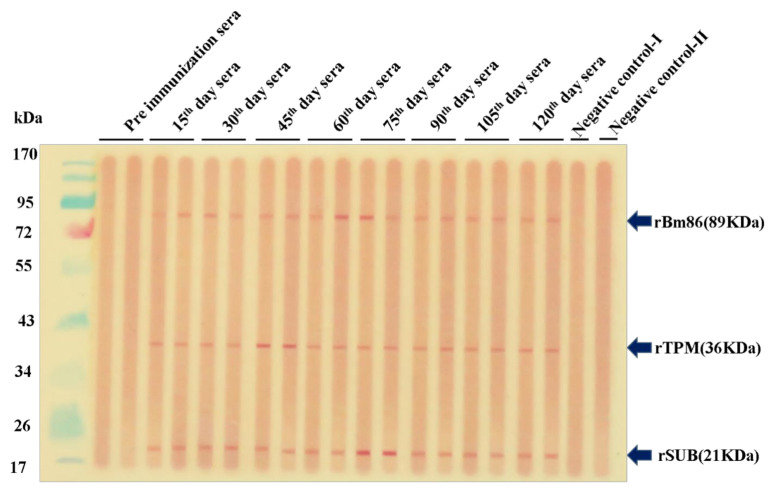
Immuno-reactivity of the rBm86 (89 kDa), rHaTPM (36 kDa), and rHaSUB (21 kDa) proteins against sera collected from immunized calves on different days using multi-screen Western blotting apparatus.

The co-immunization of animals using the rBm86, rHaTPM, and rHaSUB proteins stimulated the production of specific antibodies as early as from the 15th day of primary immunization (DPI) and was observed until end of the experiment (140th day DPI) through a Western blot [57]. No reaction was noted against the negative control-I (fetal calf serum) and negative control-II (pooled sera sample of control animals) to any of the proteins (Figure 3). 

**Figure 4:**
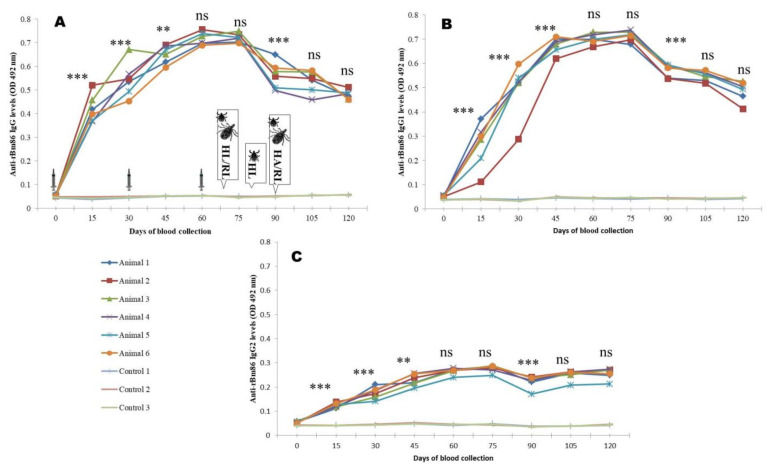
rBm86-induced IgG (**A**), IgG1 (**B**), and IgG2 (**C**) responses in calves co-immunized with recombinant proteins. HL—*H. anatolicum* larvae; RL—*R. microplus* larvae; HA—*H. anatolicum* adults; Ns—not significant; ** *p <* 0.01; *** *p <* 0.001 in comparison to the previous day’s responses. Images of syringes imply days of immunization, while images of ticks indicate days of challenge.

**Figure 5:**
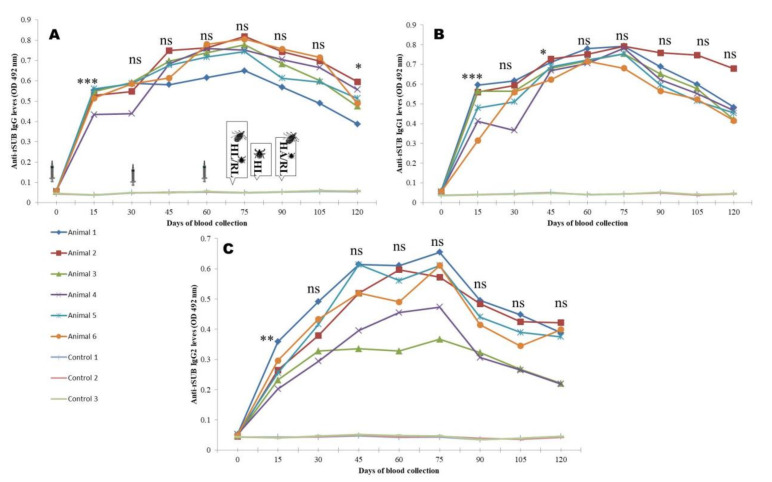
rSUB-induced IgG (**A**), IgG1 (**B**), and IgG2 (**C**) responses in calves co-immunized with recombinant proteins. HL—*H. anatolicum* larvae; RL—*R. microplus* larvae; HA—*H. anatolicum* adult; ns—not significant; * *p <* 0.05; ** *p <* 0.01; *** *p <* 0.001 in comparison to the previous day’s responses. Images of syringes imply days of immunization, while images of ticks indicate days of challenge.

**Figure 6:**
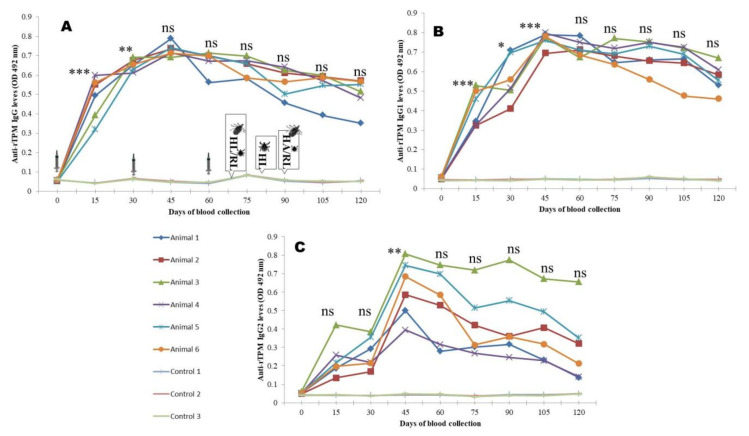
rTPM-induced IgG (**A**), IgG1 (**B**), and IgG2 (**C**) responses in calves co-immunized with recombinant proteins. HL—*H. anatolicum* larvae; RL—*R. microplus* larvae; HA—*H. anatolicum* adult; ns—not significant; * *p <* 0.05; ** *p <* 0.01; *** *p <* 0.001 in comparison to the previous day’s responses. Images of syringes imply days of immunization, while images of ticks indicate days of challenge.

The peak IgG response was 14–15, 14.1–16.1, and 13.02–16.4 times higher (*p <* 0.0001), respectively, in animals immunized by rBm86, rHaTPM, and rHaSUB in comparison to the control and maintained until the first challenge. Although, a decrease in the antibody response was noted at later stages, the antibody response was significantly (*p <* 0.001) 8.16–9.1, 6.8–11.2, and 6.8–10.5 times higher, respectively, in comparison to the control animals. Similarly, the highest (*p <* 0.0001) IgG1 response of 15.1–16.4, 14.0–16.1, and 15.1–17.6 times to rBm86, rHaTPM, and rHaSUB, respectively, was recorded and maintained until the first challenge experiment. Thereafter, a decreasing trend (*p <* 0.001) was noted until the end of the experiment. A high level of animal-to-animal variation in the IgG2 response was recorded following immunization of animals by rHaTPM and rHaSUB. For rBM86 and rHaSUB, the maximum response was 5.5–6.1 and 8.1–14.5 times higher in comparison to the mean response in the control animals. However, in the case of rHaTPM, a significant (*p <* 0.05) variation of 3.06–9.5 times in the immunized group of animals on the 15th DPI with a maximum variation of 2.7–13.2 times on the 120th DPI was noted (Figure 4, Figure 5 and Figure 6). The IgG antibody titer on the 90th DPI against rBM86 and rHaTPM was 1: 102, 400 and 1: 51,200, whereas the maximum titer against rHaSUB was 1: 64,000 on the 75th DPI. Overall, a significant difference (*p <* 0.0001) in the IgG, IgG1, and IgG2 responses was recorded in the immunized animals in comparison to the control animals. From the ratios between the IgG to IgG1 and IgG2 responses it was observed that, initially, the IgG response was much higher as compared to IgG1 and IgG2. A similar trend was recorded when the ratio between the IgG1 to IgG2 responses was compared (Supplementary Table S3). 

### 3.5. Protective Efficacy

The experimental calves were agile, feed and water intakes were normal, body temperatures were within the normal range, and no tick-borne infections were noticed in blood smear examinations throughout the experimental period of 140 days.

Against *H. anatolicum*: Within 48 h of release, ticks started feeding on animals. Most of the engorged larvae dropped within 2 days of initiation of dropping from the control animals, while dropping of engorged larvae from the immunized animals lasted for 4–6 days after initiation. Significant (*p <* 0.05) reductions in the dropping (47.26%) and moulting (76.09%) percentages of larvae fed on the immunized group of animals in comparison to larvae fed on the control animals were noted. The comparative post-challenge parameters, viz, the percentage reduction in the number of larvae (DT%), the moulting percentage (MO%), and the efficacy against larvae (E%), were determined as 49.1%, 73.7%, and 86.6% after the first challenge, while after the second challenge, they were 45.5%, 78.4%, and 88.2%, respectively (Table 1A). The mean efficacy of immunization of animals against *H. anatolicum* larvae was 87.4%.

The feeding period of female ticks was in the range of 5–12 days in the two groups of animals. Unlike larvae, there was no statistical difference in the mean number of adult ticks dropped from the immunized and control groups of animals. However, a significant (*p <* 0.001) reductions of 29.9% in the mean engorgement weight and egg masses (66.8%) laid by ticks fed on the immunized group of animals in comparison to ticks fed on the control group of animals were observed. Eighty-three ticks dropped from the immunized animals were poorly fed, while the forty-four ticks dropped from the control animals were completely fed and normal (Supplementary Figure S4). Consequently, the DT%, DR%, DO%, RF%, and E% were determined as 5.4%, 29.9%, 66.8%, 56.2%, and 86.2%, respectively (Table 1). The mean immunization efficacy was calculated as 86.02% against *H. anatolicum* larvae and adults together.

Against *R. microplus*: The challenged larvae started feeding within 48 h of release. After 18–19 days of feeding, engorged adults started to drop from all the animals. Significant reductions in the mean number of ticks dropped (*p <* 0.0001), mean engorgement weight (*p <* 0.005), and mean egg masses (*p <* 0.0001) of ticks collected from immunized animals in comparison to the control group of animals were noted. Consequently, due to the significant reduction in egg masses, the reproductive index was significantly (*p <* 0.0001) reduced by 35.8%. The DT%, DR%, DO%, RF%, and E% were determined as 63.0%, 26.0%, 53.0%, 35.0%, and 88.9%, respectively. Similar observations were recorded forthe second challenge with minor changes, viz., 52.8%, 20.0%, 49.8%, 34.0%, and 84.6% for the corresponding entomological parameters, respectively (Table 2). As observed in the case of *H. anatolicum* adults, the mean engorgement weight of eighty-three ticks fed on immunized animals was 276.3 mg, whereas the mean engorgement weight of forty-four ticks fed on control animals was calculated as 394.3 mg (Supplementary Figure S4). The mean efficacy of immunization of animals against *R. microplus* was 86.8%.

## 4. Discussion

The concept of immunization of hosts against ticks has long been established, and two vaccines, TickGARD^TM^ and Gavac^TM^, were commercialized [29,58] and adopted in some Latin American countries and provided significant benefits over the use of chemical acaricides [29]. However, the cost and adaptability of the veterinary vaccine technology in wide geographical areas are the two very important attributes. After the breakthrough discovery and development of the *R. microplus* vaccine, attempts have been made to evaluate the protective efficacy of the vaccine against different strains of *R. microplus* with highly variable, nil to 91%, efficacies against field infestations recorded [55]. Variation in the amino acid sequence amongst the different strains was identified as one of the reasons [55,59]. 

The dairy production systems in Asia and Africa are suffering from multiple tick infestations, and thus, the commercial success of the BM86 vaccine targeting only *R. microplus* could not be achieved. For example, in an immunization trial using the Gavac^TM^ vaccine, efficacies of 44.5% against *R. microplus* IVRI-I and 25.1% against *H. anatolicum* IVRI–II strains were observed [35]. For the development of a successful cross-protective vaccine, several targets were identified with a limited cross-protective efficacy [60,61]. Parizi [60] and Willesden [62] are of the opinion that an anti-tick vaccine containing multiple antigens is expected to be more effective than a single-antigen vaccine. Schetters and Jansen [44] provided a significant lead by achieving 97% cross-protection against *R. annulatus* following co-vaccination of animals with yeast-expressed Bm86 and *E. coli*-expressed SUB antigens of *R. microplus*. Accordingly, in this immunization trial, conserved antigens were selected to have a higher efficacy against homologous challenge infestations with an aim to conduct an additional trial against heterologous challenges if communization efficacy is improved significantly against homologous challenges. 

Multiple-sequence analysis of the IVRI-I *Bm86* gene revealed a conserved region of 192 amino acid, and it has 95.6 to 99.8% and 93.2 to 99.5% identity with nucleotide and predicted amino acid sequences between 68 Indian field isolates. The polymorphism might be because of diversity in the tick population adapted from the different geographic and climatic conditions of India. The diversity is also observed in the entropy plot where changes in 30 sites were recorded in 192-amino-acid-long conserved *BM86* gene sequences. The sequence identity matrix analysis showed that the IVRI-I Bm86 protein has a sequence identity of 93.2% (6.76% divergence) and 92.7% (7.22% divergence) with the Yeerongpilly (TickGARD) and Camcord (Cuba) (GAVAC) vaccine strains, respectively [49]. The sequence divergence data validate the earlier observation in which 44.5% and 25.1% efficacies against *R. microplus* (IVRI-I strain) and *H. anatolicum* (IVRI-II strain), respectively, were recorded in a pen trial using the commercial Cuban Bm86 vaccine [35]. The high diversity of IVRI-I Bm86 and the low efficacy of the commercial Mexican Bm86 vaccine in India show that there is a strong need for an Indian-specific Bm86 peptide vaccine consisting of immunogenic epitopes. In contrast, the SUB and TPM homologues of *H. anatolicum* showed very high levels of identity within the species and among tick species. The variationsin the predicted amino acid levels were 0–1.9% and 0.4–1.4%, respectively, among the different field isolates. The present observations are in agreement with previous studies [36,37] where 98.7–99.9% and 97.6–99.4% similarities in deduced amino acid sequences of TPM and SUB, respectively, in various *H. anatolicum* Indian field isolates were recorded. Moreover, very high levels of sequence identity of 99.6 and 93.2% in the TPM and SUB genes of *H. anatolicum* with *R. microplus* were observed in the present study, which supports the idea of the development of a cross-protective vaccine using the SUB and TPM proteins [36]. Both the genes or their homologues were reported in mammals with a sequence identity of less than 46% with*B. taurus/B. indicus* Akirin/*SUB* and less than 58% with *B. taurusTPM1* or *TPM2* to the IVRI II strain; the chance of developingan autoimmune problem in the host is minimal because it is expected that the antibody response would be primarily directed against non-self-epitopes, and immunization with intracellular proteins has been reported to be effective in ticks and in other invertebrate organisms [63].

The Shannon entropy value [64] measures the variation in or diversity of nucleotides or amino acids in aligned sequences.The variation in amino acids alters the protein three-dimensional structure and function. Analysing the entropy plots, Bm86 showed the maximum (30), TPM (8), and minimum (6) in SUB proteins of Indian field isolates. The diversity in the Bm86 gene sequence in *B. microplus* isolates confirms that Bm86 alone may not be a suitable candidate for the development of a vaccine against *R. microplus*. However, its significant identity with the SUB and TPM sequences justifies its inclusion in the multi-antigen immunization study. In a separate experiment, we tested multi-epitope-based peptide vaccines, avoiding highly variable amino acids across the different field isolates, and post-challenge entomological data are to be generated.

For the effective management of ticks, it is necessary to manage all the stages so that comprehensive control can be achieved. Since each and every stage of the parasite has a specific requirement to survive, there are many molecules which are either up- or down regulated. So, it is worthy to quantify the gene expression profiles of the targeted genes in different stages of ticks. A significant (*p <* 0.0001) increase in the fold change inthe expression of *BM86* was observed in eggs, males, and partially fed and fully fed females in comparison to larvae, while in eggs, the difference was at a *p <* 0.05 level. Similarly, Bastos [65] quantified the *BM86* expression profile in different stages of *R. microplus*, and high-level expression in partially engorged females and a low expression in unfed larvae were reported. Previously, in the laboratory, the expression profile of the SUB gene in *H. anatolicum* was determined, and the maximum expression in eggs, frustrated females, and the lowest in adults was noted [36]. For the TPM gene expression, the maximum was recorded in fed nymphs, females, males, and in unfed adults [37]. As the targeted genes were expressed significantly in all the stages, these genes were further qualified as possible vaccine candidates. 

To assess the protective efficacy of the selected molecules, recombinant proteins were generated in eukaryotic expression systems with an aim to achieve better efficacy. The targeted genes were cloned separately in a pKLAC2 vector in frame with the α-mating factor (α-MF) secretion leader sequence. The expression kinetics of the targeted proteins were worked out, and the desired proteins of the expected sizes of ~89 kDa, 21 kDa, and 36 kDa were obtained. Though the expression level in *K. lactis* was satisfactory, column-based protein purification has the limitation of exhaustion, and the column cannot be reused. It is necessary to standardize the scale-up conditions and purification steps for the economical production of the targeted antigens. 

The immunization efficacy is dependent on many factors, for example, the dose, site of inoculation, adjuvant used, etc. [66,67]. As no separate experiment was conducted to optimize the dose of the Bm86 antigen, the literature was scanned, and the dose of 100 µg was selected [68,69,70]. For a strong antibody response against the TPM protein, Manjunathachar et al. [37] standardized a total dose of 300 µg in three equal doses. Similarly, Kumar et al. [36] reported a strong antibody response following immunization of animals with a 300 µg total dose of the SUB protein. Accordingly, a co-immunization trial was conducted on calves using 100 µg of each antigen with an equal volume of Montanide ISA 50V2 per dose. 

The role of the humoral antibody response for conferring immunity against challenge infestations has been correlated repeatedly [71], and accordingly, the antibody response was monitored by indirect ELISA. Kemp [72] and Penichet [73] reported that IgG1 alone or perhaps with the aid of a complement is enough to damage the tick gut. Tellam [74] elaborated that the IgG1 subclass dominates over IgG2 or IgM and playsa crucial role in altering the physiology of the challenged ticks. Accordingly, to investigate the mechanism of immunity against challenge infestations following co-immunization, the IgG, IgG1, and IgG2 responses were characterized. A significantly high anti-Bm86 IgG response in all the animals with a maximum titer of 1:102,400 was noted. The IgG1 response dominated over the IgG2 response. For the rHaTPM antigen, the IgG2 response was comparatively lower in comparison to IgG and IgG1, and maximum titer of 1:51,200 was noted. For the rHaSUB antigen, the IgG1 response dominated over IgG2, and a maximum IgG titer of 1:64,000 was noted. Previously, in our laboratory, while conducting immunization trials using the Haa86, SUB, TPM, cathepsin, and calreticulin antigens, a similar trend in the IgG, IgG1, and IgG2 antibody response was recorded [36,37,41,75]. Similarly, in immunization trials using the rBM86 antigen, the antibody response was in the trend of IgG > IgG1 > IgG2 [64,65]. The Bm86 antigen induced an immune response in order of IgG > IgG1 > IGg2 on the 15th and 75th day post-primary immunization (PPI), whereas on the 120th day PPI, it was predominantly IgG2 over IgG and IgG2. A similar type of antibody trend was reported earlier using Bm86 [76], the Haa86 antigen [63,77]. The SUB antigen induced IgG > IgG1 > IgG2 on the 15th day and the 120th day PPI. Interestingly, on the 75th day PPI, the IgG2 antibody was predominant over IgG and IgG1, as reported previously [41]. SUB is not a secretory protein and so is not available in salivary secretion to naturally stimulate the immune system of the host. Accordingly, the absence of a post-challenge hike in the antibody response in animals against the SUB antigen was observed [78,79]. The TPM antigen induced predominantly IgG over IgG1 and IgG2 on the 15th day PPI, whereas on the 75th and 120th day PPI, a higher level of IgG2 than IgG and IgG2was noted, as reported in [37]. Isotype switching is heavily influenced by cytokines in the immediate micro-environment of the activated B-cells [80]. Therefore, after challenges due to tick salivary secretion, it might be changed in the cytokine profile of the host. This kind of antibody response was observed in previous studies using the SUB [41], Haa86 [75,79] and Bm86 [81].

Parizi [59] monitored the immune response following the immunization of animals with vitelline-degrading cysteine endopeptidase (rVTDCE), the *Boophilus* yolk pro-cathepsin (BYC) of *R. microplus*, and the glutathione S-transferase of *H. longicornis* (GST-Hl). More than 10, 2, and 6 times increases in the antibody titers for rVTDCE, rBYC, and rGST, respectively, after 78 days of immunization compared to the pre-immunization values were recorded. The immune response recorded in the present study was initiated at a very early stage and was continued until 120 DPI against all the antigens. However, the peak antibody response showed a declining trend in the absence of natural stimulus, a limitation of vaccines based on concealed antigens [55]. 

The experiment by Hatfield [82] and Vaughan [33] indicated that intracellular proteins of arthropods can be targeted by antibodies. Accordingly, some of the intra-cellular proteins, such as SUB, TPM, cytoplasmic nucleotidase, interfase cytoplasmic foci protein 45, heat shock, and ribosomal proteins, were tested and found to be effective against ticks and other invertebrate organisms [2,34,36,78,79]. The intracellular availability of the antibody might be dependent upon its concentration outside the cell. The mechanisms by which antibodies are transported across arthropod cell membranes and interact with antigens are not completely understood and need further research [34]. Tropomyosin (TPM), an actin regulator protein, is required for the proper contraction of muscles and is necessary for important physiological processes. The antibodies binding to different muscle tissues in ticks might hamper the physiological functions of *Hyalomma* ticks leading to loss of important physiological functions such as blood feeding, egg laying process, etc. Subolesin is involved in the regulation of gene expressions through interactions with intermediate regulatory proteins such as GI, GII, 14-3 beta, and other yet to be identified [2,69,83]. These intermediate proteins interact with NF-kB, bind DNA, or remodel chromatin to regulate gene expressions. A similar trend in the antibody response of SUB and TPM antigens was recorded, as reported previously [78,79].

Both the immunized and control animals were challenged with the larvae of *R. microplus* and the larvae and unfed adults of *H. anatolicum*. Interestingly, the *H. anatolicum* larvae and adults attached immediately after release on the control animals, but delayed attachment to the immunized animals was observed, and a mean efficacy of 86.02% against *H. anatolicum* was noted. The delayed feeding may be due to the localization of the SUB and TPM antigens in the salivary glands and in the muscle fibers of different organs. A 29.9% reduction in the mean engorgement weight of ticks fed on the immunized group of animals in comparison to ticks fed on the control group of animals was noted. Consequently, a significant 66.8% reduction in the mean egg masses laid by the ticks dropped from the immunized group over the control group of animals was observed. Merino [83] recorded high anti-rSUB antibody titers following the first and subsequent immunizations linked with 42% protection against the challenged infestations. The immunization of calves with rBmSu resulted in anIgG1-mediated efficacy equal to 44.0% and 37.2% after the first and second challenges, respectively [41]. The immunization of calves by the yeast-expressed rSUB of *H. anatolicum* conferred an efficacy of 86.2% against adults, 88.9% in the first, and 84.6% in the second larval challenge infestations. Similar types of results were observed in *B. indicus-*immunized animals using the *R. appendiculatus* SUB antigen with a 47% efficacy against *R. appendiculatus* and a 50% efficacy against *A. variegatum*, whereas in cross-bred cattle, *R. appendiculatus* SUB immunization gave 90%, 89%, and 51% against challenge infestations against *R. appendiculatus*, *A. variegatum*, and *R. decoloratus*, respectively [84]. Another study using the *H. anatolicum* SUB-protein for immunization gave 65.4% and 54.0% protection against *H. anatolicum* and *R. microplus* challenge infestations in calves [36]. In the case of the rTPM protein, 63.7% protection against larvae and 66.4% protection against adults of an *H. anatolicum* challenge infestation were reported [37]. In all the above experiments, the high antibody titer was positively correlated with the vaccine efficacy. 

The reduction in the mean egg masses may be due to the binding of the SUB and TPM antibodies to specific antigens, which are present in muscle fibers around ovary and ovarian follicles, leading to production of less egg masses for *H. anatolicum* fed on immunized animals. Previously, Manjunathachar et al. [37] reported a 63.7% and 66.4% efficacy against *H. anatolicum* larvae and adults, respectively, following immunization of animals using rHaTPM alone. Similarly, an immunization trial using the rHaSUB protein provided 65.4% protection against an *H. anatolicum* challenge infestation [36]. The data obtained in the present investigation and the reports available in the literature clearly indicate that co-immunization with three antigens significantly (more than 20%) improves the immunization efficacy against challenge infestations. In the current co-immunization trial, efficacies of 88.9% in first and 84.6% in the subsequent challenge infestations (mean 86.8%) against *R. microplus* were noted, and the data are comparable with other experiments conducted in different countries. For example, efficacies of 91% [74], 75% [55,59,85], and 51% [55,59,86,87] were presented from Cuba, Australia, and Mexico, respectively. The reduction inthe mean number of ticks may be due to the binding of anti-BM86 antibodies to the Bm86 antigen present in the gut tissue and the complement-mediated lysis of the gut (some ticks turned red in colour due to gut damage) [88]. Significant reductions of 53.50% and 49.8% in the mean egg masses of ticks dropped from the first and subsequent challenge infestations of the immunized group of animals in comparison to ticks dropped from the control group of animals were noted. The reduction in egg laying may be due to the effect of the BM86 antibodies in the blood digestion process in the gut, thus inhibiting the rate of conversion of blood meal into egg masses in ticks fed on the immunized group of animals in comparison to ticks fed on the control group of animals. Similar reductions in the egg laying capacity, reductions in the number of engorged ticks, and severe gut damage were reported previously when animals were immunized with the BM86 antigen [78,89,90,91]. In conclusion, the present study provided strong evidence of the potential of the co-immunization of animals with conserved multifunctional antigens for achieving better protection against multi-tick infestations.

## Figures and Tables

**Table 1:** (**A**) Mean protective efficacy of recombinant proteins against challenge infestations of *H. anatolicum* IVRI-II larvae (Mean ± SE). (**B**) Mean protective efficacy of recombinant proteins against challenge infestations of *H. anatolicum* IVRI-II adults (Mean ± SE).

(A)														
Challenge Infestations		Group		No. of Engorged Larvae Dropped		No. of Larvae Moulted to Nymphs			DT%		MO%		E%	
First larval Challenge		Immunized		198.5 ± 11.11 *		99.5 ± 10.75 *			49.05		73.74		86.62	
		Control		389.66 ± 64.67		379.0 ± 66.68								
		Reduction%		49.05		73.4								
Second larval Challenge		Immunization		247 ± 13.42 **		96.5 ± 3.96 **			45.47		78.44		88.24	
		Control		453 ± 20.30		447.66 ± 20.11								
		Reduction%		45.5		78.5								
**(B)**														
**Group**	**Tick Dropped/Animal** **(Mean ± SE)**		**Tick wt. (g) (Mean ± SE)**		**Egg wt.(g) (Mean ± SE)**		**RI(Mean ± SE)**	**DT%**		**DR%**	**DO%**	**RF%**		**E%**
Immunization	13.83 ± 0.3073		0.2763 ± 0.007 **		0.105 ± 0.006 **		0.3511 ± 0.01 **	5.4		29.9	66.81	56.2		86.2
Control	14.67 ± 0.3333		0.3943 ± 0.007		0.3164 ± 0.007		0.8019 ± 0.030							
Reduction%	5.7		29.9		66.8		56.2							

Significant at * *p <* 0.05 and ** *p <* 0.001 in comparison to the control group.

**Table 2:** Mean protective efficacy of immunization of animals against challenge infestations of *R. microplus* IVRI-I larvae (Mean ± SE).

Group	Tick Dropped/Animal (Mean ± SE)	Tick wt. (g) (Mean ± SE)	Egg wt.(g) (Mean ± SE)	RI (Mean ± SE)	DT%	DR%	DO%	RF%	E%
First Larval Challenge									
Immunization	27.33 ± 1.054 *	0.1065 ± 0.004 **	0.0498 ± 0.002 *	0.4761 ± 0.013 *	63	26	53	35	88.9
Control	74 ± 3.606	0.1444 ± 0.007284	0.1071 ± 0.006013	0.7417 ± 0.00515					
Reduction%	63.06	26.2	53.50	35.8					
Second Larval Challenge									
Immunization	46.17 ± 1.108 *	0.1172 ± 0.0020 *	0.05506 ± 0.001639 *	0.476 ± 0.01615 *	52.8	20.4	49.8	34	84.6
Control	98 ± 1	0.1473 ± 0.001866	0.1097 ± 0.002133	0.734 ± 0.006956					
Reduction%	52.8	20.4	49.8	35.1					

Significant at * *p <* 0.0001 and ** *p <* 0.001 in comparison to the control group.

## Data Availability

Data sets used and/or analyzed during the present study are available from the corresponding authors on reasonable request.

## Supplementary Materials

The following supporting information can be downloaded at: https://www.mdpi.com/article/10.3390/pathogens12030433/s1, Figure S1: Phylogenetic analysis of different field isolates of *Rhipicephalus microplus* through maximum-likelihood method using the Jones–Taylor–Thornton (JTT) model on (1000 pseudo replicates) conserved Bm86 amino acid with worldwide published vaccine strain sequences (bootstrap values lower than 60% removed); Gujarat sequences were arranged in a group (red-coloured box). Figure S2: Entropy plot of (A) conserved Bm86, (B) subolesin (SUB), and (C) tropomyosin (TPM) sequences of Indian field isolates. Figure S3: Phylogenetic analysis of different isolates of *Hyalomma anatolicum* along with other ticks, arthropods, and mammals on deduced amino acid sequences of (A) subolesin (statistical method:maximum-likelihood; model: Jones–Taylor–Thornton (JTT) + gamma distribution (+*G*) with 5 rate categories; bootstrap value:500; neighbour-join and BioNJ algorithms) and (B) tropomyosin (statistical method: maximum-likelihood; model: Le_Gascuel_2008 (LG)+ gamma distribution (+*G*) with 5 rate categories; bootstrap value:500; neighbour-join and BioNJ algorithms) (bootstrap values lower than 60% removed). Figure S4: Representative sample of ticks dropped from immunized animals compared to control animals. (A) *Hyalomma anatolicum* female dropped from control animals; (B) *H. anatolicum* female dropped from immunized animals; (C) *Rhipicephalus microplus* female ticks dropped from control animals; and (D) *R. microplus* female ticks dropped from immunized animals. Table S1: Details of PCR primer used in the experiment. Table S2: Field samples of *R. microplus* and *H. anatolicum* collected from different parts of India. Table S3: The ratio of IgG to IgG1 and IgG2 and IgG1 to IgG2 responses against different recombinants proteins after immunization.
