# Supplementary material for: Co-Immunization Efficacy of Recombinant Antigens against Rhipicephalus microplus and Hyalomma anatolicumTick Infestations
Source: Pathogens. 2023 Mar 9;12(3):433. doi: 10.3390/pathogens12030433 (PMC10058648; doi:10.3390/pathogens12030433)
Supplement: Supplementary file 1 [file pathogens-12-00433-s001.zip › pathogens-2118738-supplementary.pdf]

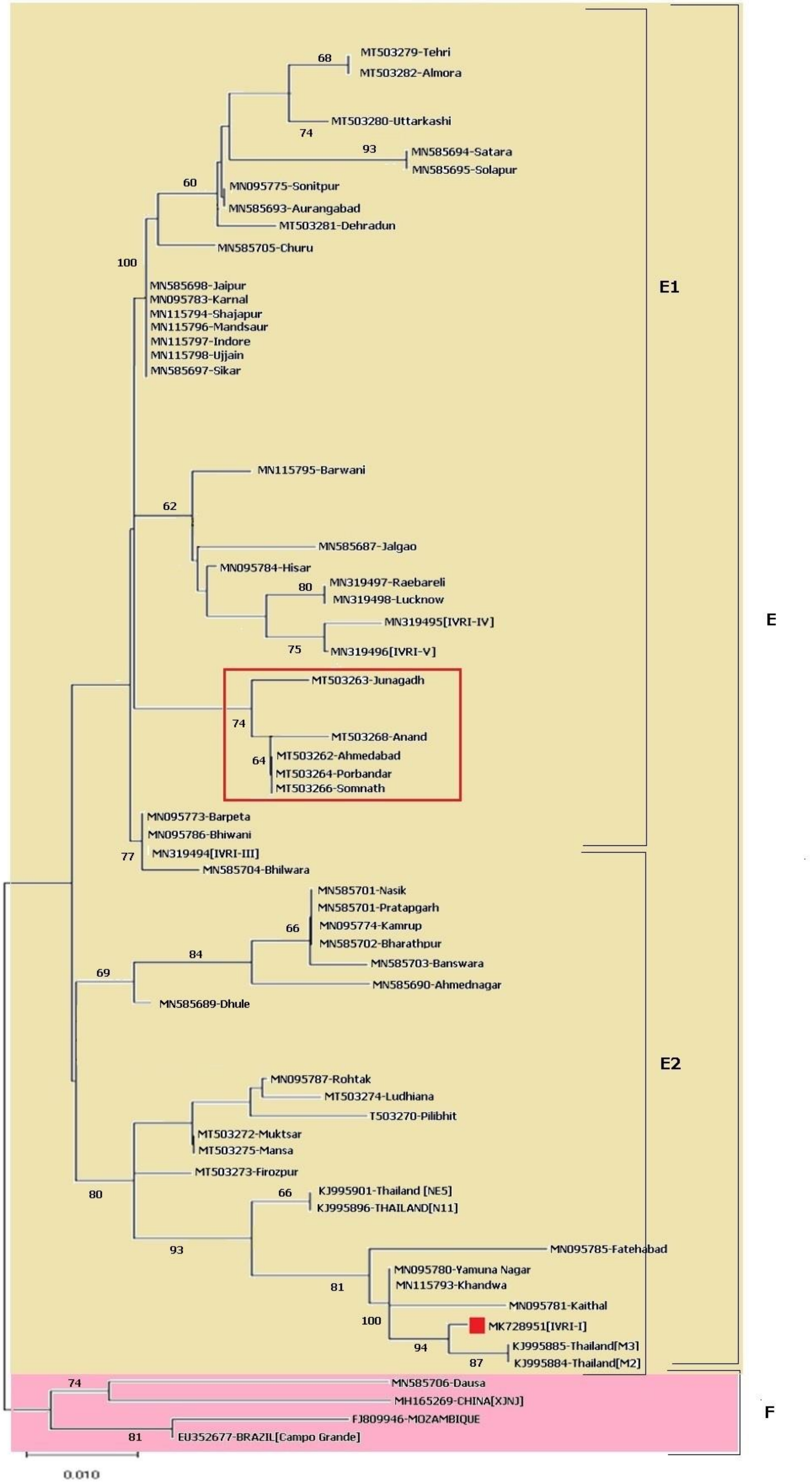

Figure S1. Phylogenetic analysis of different field isolates of *Rhipicephalus microplus* through maximum-likelihood method using Jones-Taylor-Thornton (JTT) model on (1000 pseudo replicates) conserved Bm86 amino acid with worldwide published vaccine strain sequences (Bootstrap values lower than 60% removed); Gujarat sequences were arranged in a group (red color box).

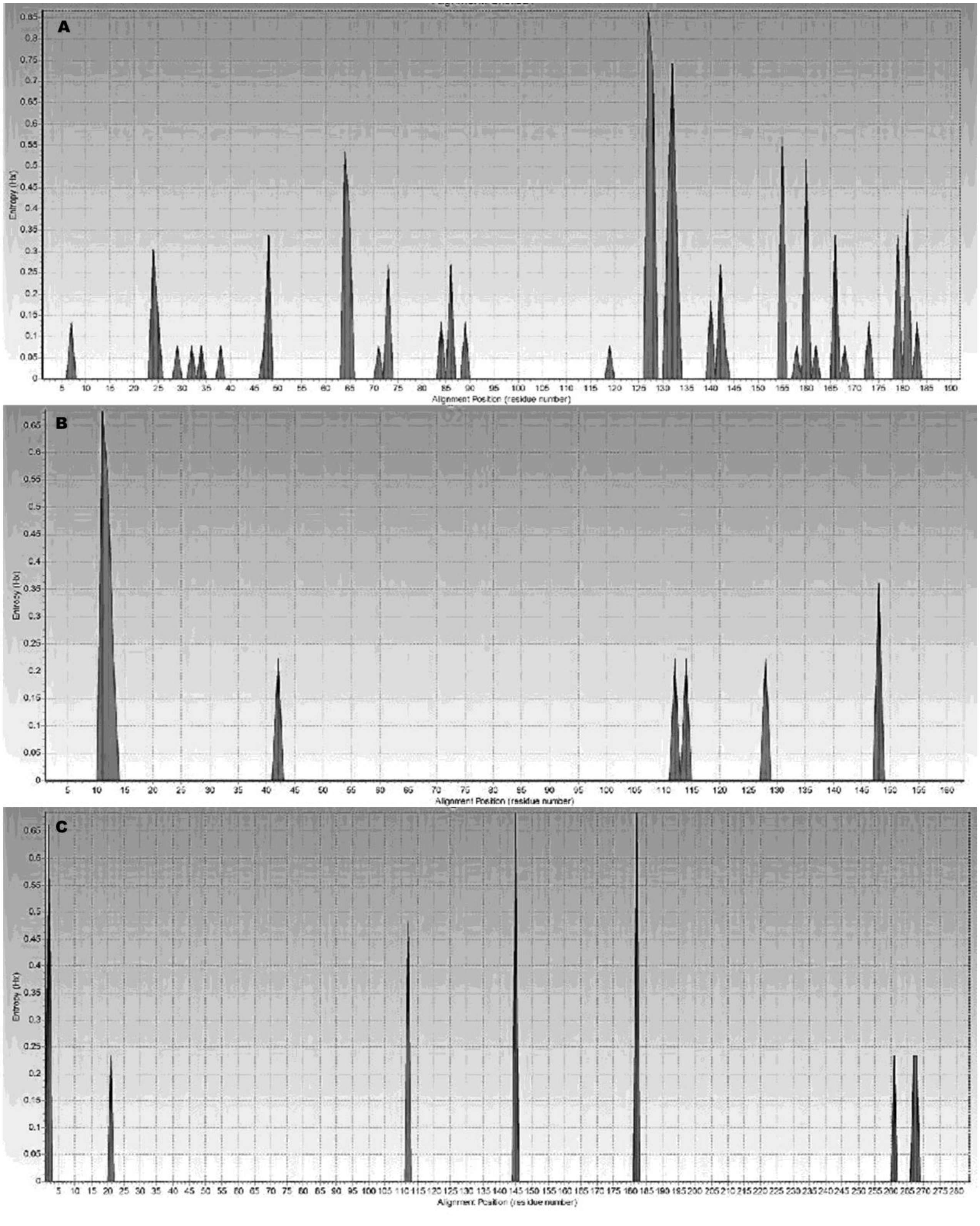

Figure S2. Entropy plot of **A.**) Conserved Bm86, **B.**) Subolesin (SUB) and **C.**) Tropomyosin (TPM) sequences of Indian field isolates.

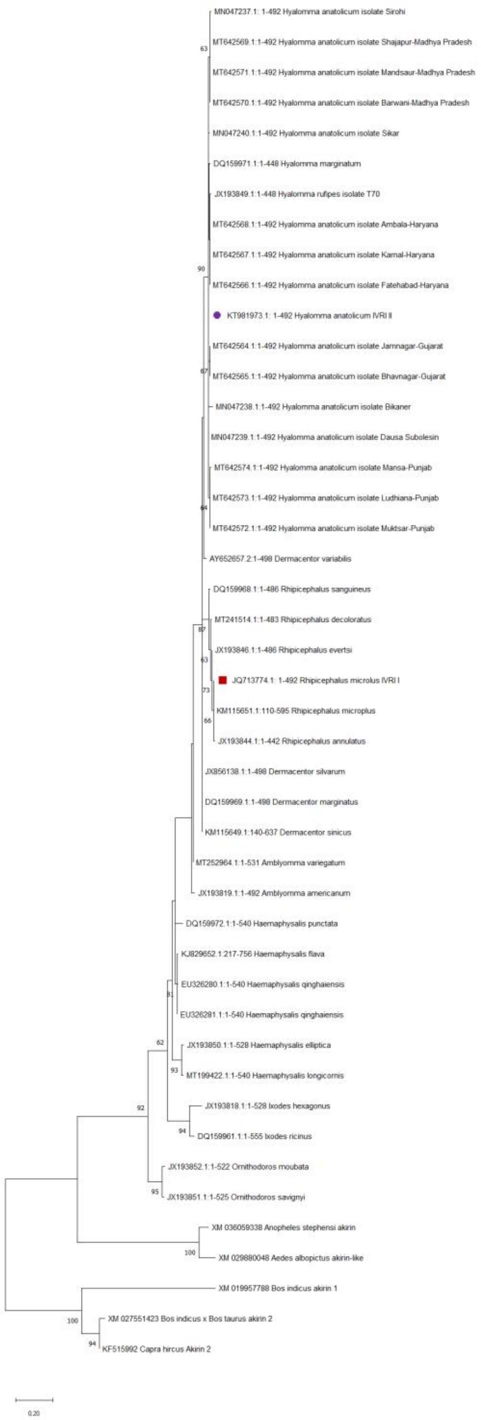

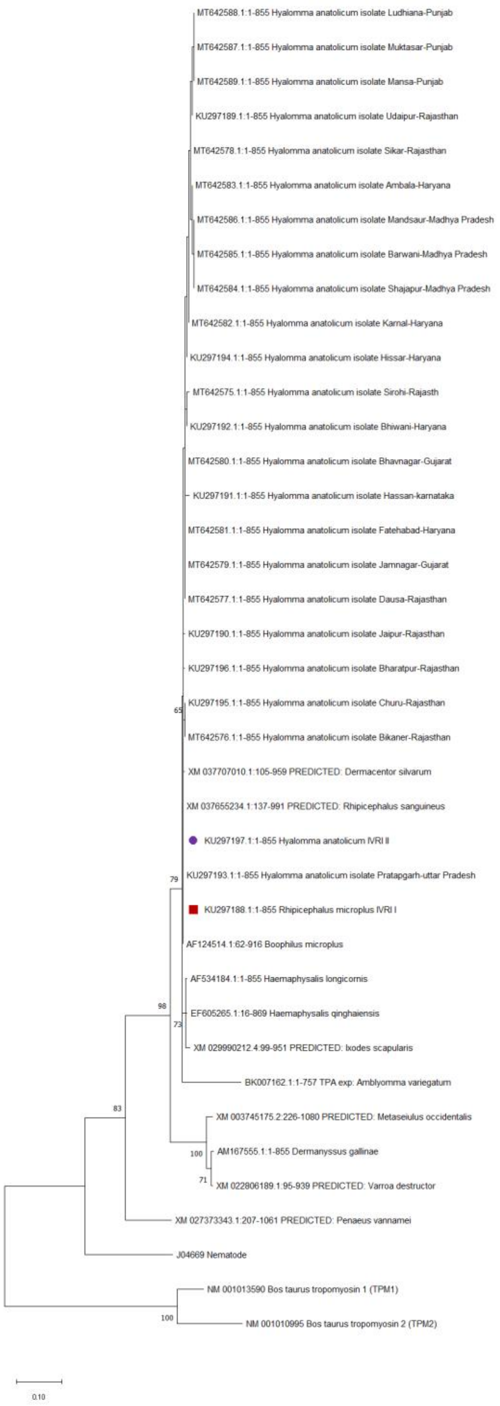

Fig. S3. A **Fig. S3. B**

Figure S3. Phylogenetic analysis of different isolates of *Hyalomma anatolicum* along with other ticks, arthropods and mammals on deduced amino acid sequences of (**A**) Subolesin (statistical Method: Maximum-likelihood; model: Jones-Taylor-Thornton (JTT) + Gamma distribution (+*G*) with 5 rate categories; bootstrap value:500; Neighbor-Join and BioNJ algorithms) (**B**) Tropomyosin (statistical Method: Maximum-likelihood; model: Le_Gascuel_2008 (LG) + Gamma distribution (+*G*) with 5 rate categories; bootstrap value:500; Neighbor-Join and BioNJ algorithms). (Bootstrap values lower than 60% removed).

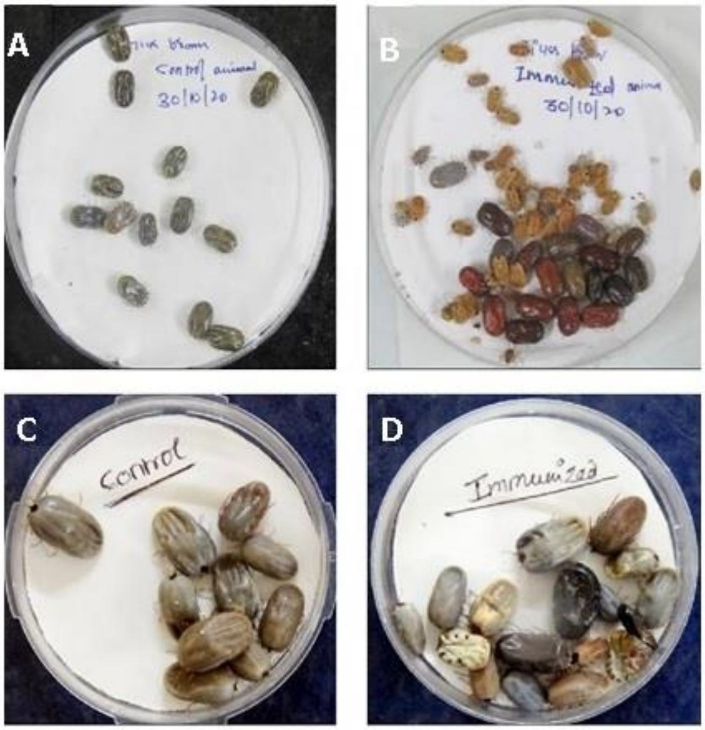

Figure S4. Representative sample of ticks dropped from immunized animals compared to control animals. **A.**) *Hyalomma anatolicum* female dropped from control animals; **B.**) *H. anatolicum* female dropped from immunized animals; **C.**) *Rhipicephalus microplus* female ticks dropped from control animals; and **D**.) *R. microplus* female ticks dropped from immunized animals.

Table S1. Details of PCR primer used in the experiment

Genes	Primer sequence (5´→3´)	Length	Amplicon size(bp)	Use
Full length Bm86 gene	Atgcgtggcatcgctttatt	20	1953	Full length
	gtttagcccaactatctttatttgacatc	22		
Bm86 gene conserved sequence	Tgcgacagtctgctcaagaat	21	578	For cloning, sequencing and gene conservation study
	Gctgcagcacttgactttcca	21		
SUB (*H. anatolicum*)	Gaaatcctacgcacccaatg	20	492	
	ttagacgagtgggtagctggc	21		
TPM (*H. anatolicum*)	atgga(g/t)gccatcaagaa(g/a)aa(g/a)atgcag	26	901	
	gagcagcggtrgaaacaacggc	22		
Bm86	aggctcgagaaaagaatgcgtggcatcgctttgttc	36	2	For expression of gene in eukaryotic system (Primers with vector flanking region)
	Agggcggccgcttatgcataatctggaacatcatatggataatacaacgatgctgcggtgac	62		
TPM	aggccatggaaaagaatggaggccatcaagaaaaagatgcag	42	911	
	Aggggatccttatgcataatctggaacatcatatggataatagtagccagtgagttcggagaaggt	66		
SUB	aggctcgagaaaagaatggcttgtgcgacattaaag	36	548	
	Agggcggccgcttatgcataatctggaacatcatatggataatacgacaaatagctgggcgt	62		
Bm86	Gaggatcgtgttttggaagc	20	133	For gene quantification (qPCR)
	Tcctgagcacgtgttttgac	20		
EF 1-alpha	cgtctacaagattggtggcatt	22	109	
	Ctcagtggtcaggttggcag	20		
GAPDH	agt ccaccggcg tcttcctca	21	123	
	gtgtggttcacacccatcacaa	22		

Table S2. Field samples of *R. microplus* and *H. anatolicum* collected from different parts of India

States	Districts	Total samples
	R. microplus collection areas	
Assam	Nagaon, Barpeta, Kamrup, Sonitpur, Morigaon and Dibrugarh	6
Haryana	Panipat, Kurukshetra, Yamuna Nagar, Kaithal, Ambala, Karnal, Hisar, Fatehabad, Bhiwani, Rohtak and Sonipat	11
Rajasthan	Alwar, Sikar, Churu, Jaipur, Pratapgadh, Bhilwara, Banswara, Udaipur, Chittorgarh, Dungarpur, Dausa and Bharatpur	12
Punjab	Muktsar, Ludhiana, Mansa, Moga and Firozpur	5
Gujarat	Ahmedabad, Jungadh, Porbandar, Jamnagar, Somnath, Bhavnagar and Anand	7
Uttar Pradesh	IVRI-I, IVRI-III, IVRI-IV, IVRI-V, Lucknow, Raebareli and Pilibhit	7
Uttrakhand	Haridwar, Newtehri, Uttarakashi, Dehradun, Almora and Mukteshwar	6
Maharashtra	Jalgaon, Nashik, Dhule, Ahmadnagar, Raigarh, Pune, Aurangabad, Satara and Solapur	9
Madhya Pradesh	Khandwa, Shajapur, Barwani, Mandsaur, Ujjain and Indore	6
	H. anatolicum collection areas	
Rajasthan	Sikar, Bikaner, Dausa and Sirohi	4
Gujarat	Jamnagar and Bhavnagar	2
Haryana	Fatehabad, Karnal, Ambala	3
Madhya Pradesh	Shajapur, Barwani and Mandsaur	3
Punjab	Muktsar, Ludhiana and Mansa	3

Table S3. The ratio of IgG to IgG1 and IgG2; and IgG1 to IgG2 responses against different recombinants proteins after immunization

Days of Post Primary Immunization (PPI)	Immunization with rBm86			Immunization with rTPM			Immunization with rSUB		
	IgG:IgG1	IgG:IgG2	IgG1:IgG2	IgG:IgG1	IgG:IgG2	IgG1:IgG2	IgG:IgG1	IgG:IgG2	IgG1:IgG2
15th day PPI	1.5:1	3.3:1	2.1:1	1.17:1	2.05:1	1.75:1	1.07:1	1.95:1	1.81:1
75th day PPI	1:1	2.6:1	2.5:1	0.93:1	1.51:1	1.6:1	0.99:1	1.3826:1	1.3844:1
120th day PPI	0.98:1	1.8:1	1.9:1	0.89:1	1.67:1	1.8:1	1.03:1	1.49:1	1.44:1
